# *Foundations* for fairness in digital health apps

**DOI:** 10.3389/fdgth.2022.943514

**Published:** 2022-08-30

**Authors:** Teodora Sandra Buda, João Guerreiro, Jesus Omana Iglesias, Carlos Castillo, Oliver Smith, Aleksandar Matic

**Affiliations:** ^1^ R&D, Koa Health, Barcelona, Spain; ^2^ ICREA, Barcelona, Spain; ^3^ Department of Technologies of Information and Communication, Universitat Pompeu Fabra, Barcelona, Spain

**Keywords:** digital mental health, algorithmic fairness, case study, undesired bias, algorithmic discrimination

## Abstract

Digital mental health applications promise scalable and cost-effective solutions to mitigate the gap between the demand and supply of mental healthcare services. However, very little attention is paid on differential impact and potential discrimination in digital mental health services with respect to different sensitive user groups (e.g., race, age, gender, ethnicity, socio-economic status) as the extant literature as well as the market lack the corresponding evidence. In this paper, we outline a 7-step model to assess algorithmic discrimination in digital mental health services, focusing on algorithmic bias assessment and differential impact. We conduct a pilot analysis with 610 users of the model applied on a digital wellbeing service called *Foundations* that incorporates a rich set of 150 proposed activities designed to increase wellbeing and reduce stress. We further apply the 7-step model on the evaluation of two algorithms that could extend the current service: monitoring step-up model, and a popularity-based activities recommender system. This study applies an algorithmic fairness analysis framework for digital mental health and explores differences in the outcome metrics for the interventions, monitoring model, and recommender engine for the users of different age, gender, type of work, country of residence, employment status and monthly income.

Systematic Review Registration: The study with main hypotheses is registered at: https://osf.io/hvtf8

## 1. Introduction

Concerns about potential bias in the application of automated systems have been increasing over time, in particular in domains that are considered to be in highly-regulated, high-risk areas (e.g., health, crime, employment or housing as per the European AI Act of 2021 (1)). However, it is far from trivial to ensure that the service, as well as the algorithms powering such service, are free from discrimination (consistently disadvantageous) for all the sensitive groups, particularly because it requires developers and designers to engage with the social, legal and ethical facets of algorithmic fairness in a given context (such as mental health); and to design and develop solutions which incorporate these values.

In its technical usage in data modeling, the word bias indicates a preference for certain kinds of models over others, and it is indeed essential for obtaining an effective model in an efficient way. There are two types of bias: desired and undesired. In the domain of digital health, the former is the type of bias that allows for recommendations to be based on evidenced factors or drivers that maximize health and/or wellbeing. The latter is the type of bias that causes recommendations to be based on gender, race, ethnicity, sexuality, age, belief, or other characteristics protected under anti-discrimination law except in narrowly defined cases related to characteristics that indeed play a relevant role, in some specific types of recommendations. Undesired bias is articulated with greater precision in the literature as “algorithmic discrimination.” This occurs when results are systematically disadvantageous against already disadvantaged and/or legally protected groups^1^ (3). While algorithmic undesired bias in general cannot be completely avoided, it is important to dedicate preventive efforts in an attempt to investigate potential disparate impact.

In this work, we assess *Foundations*,^2^ a mental health application (app) designed to help people improve and maintain their mental wellbeing. By design, *Foundations* is built on evidence-based interventions, which are meant to be effective regardless of gender, race, ethnicity and other protected attributes. It consists of a library of content grounded in science and designed by experts in the fields of Psychology and Psychiatry to help users deal with topics such as stress, poor sleep, worry and anxious thoughts, low self-esteem, among others. Nevertheless, in this paper we argue that evidence-based tools designed not to discriminate need to be regularly evaluated in practice. In this paper, we propose applying a 7-step fairness framework to evaluate:

1. whether there is any disparate impact in the response to treatment for our digital health app, *Foundations*, and
2. whether there is any undesired bias in automated algorithms, in particular, (a) in our predictive model of deterioration in well-being and depression severity (step-ups), which can be used to passively monitor our users’ symptoms, and (b) in our recommendation engine which can be used to suggest activities that are relevant for the user.

This paper is structured as follows: Section 2.1 presents some background on fairness in mental health apps and algorithm, as well as product auditing. Section 2.2 presents the app audited in this study, *Foundations*. Section 2.3 introduces the 7 step process to assess fairness in automated systems. Section 2.4 presents the details of the randomized controlled trial (RCT) previously conducted, as well as the demographics of the participants. Section 3 presents the results of applying the 7 step process, per step, for (1) evaluating fairness in *Foundations* (Section 3.1), (2) evaluating fairness of a wellbeing step-up monitoring model (Section 3.2), and (3) evaluating fairness of a recommender system (Recsys) (Section 3.3). Section 4 presents the discussion where we provide the interpretation of the results, mention limitations of this analysis and ethical considerations. Finally, Section 5 concludes the paper.

## 2. Materials and methods

### 2.1. Background

Work-related stress is the first cause of long-term sickness absence and the second reason for sickness leave shorter than four weeks in public service workers in the UK (4). Moreover, the literature has revealed that chronic exposure to hostile working conditions leads to stress (5) and several mental disorders and physical diseases, which have differential impacts on protected groups (6–9). Stress accounts for 45% of all working days lost due to poor health (Health and Safety Executive, 2016). It has also been shown that the roots of stress are more related to personal factors for females in the UK IT sector (10). Moreover, according to Health and Safety Executive (HSE), women aged between 25–54—who are likely to be juggling many roles, including worker, mother, carer for elderly parents and homemaker, experience significantly higher stress than men (11). According to WHO, workplace burnout should be approached as a multivariable phenomenon. Therefore, in creating apps for mental health and recommender algorithms to improve mental wellbeing, the literature demands that enterprises pay attention to various structural factors at both social and organizational levels.

#### 2.1.1. Fairness in mental health apps

Mobile technologies and apps for mental self-care have been supported by the WHO, in its Mental Health Action Plan 2013–2020, and by other public organizations such as the UK National Health Service (NHS). Socio-economic and gender biases have been identified in such systems, including possible digital divide problems or lack of consideration of gender differences in wellbeing, which are influenced by complex relationships between both biological and socio-economic factors (12, 13).

Some of these mobile technologies are powered by machine learning models. Such AI systems can determine that a person belongs to one of the above protected groups through proxies, such as zip codes for a specific ethnicity. Moreover, the use of these proxies for protected attributes can sometimes be intentional (see example of zip code for race in a loan eligibility system in (14)). Removing these attributes from the data, the so-called “color-blind” approach, does not reduce risks of algorithmic discrimination, and instead can make things worse, by hampering detection and mitigation efforts (15). In addition, some of these technologies are powered by recommender systems, which can be biased for different salient groups. Specific recommendations could be considered biased (due to undesired discrimination) when recommended more to one group than another. For example, if in similar contexts an app routinely recommends women an activity such as taking cooking lessons while recommending to men that they practice an outdoors sport, this would be reinforcing stereotypical gender roles. This sort of problems affect many information access systems, including search engines and recommender systems.

#### 2.1.2. Algorithmic and product auditing

A common response to the concerns about the application of automated systems has been to codify ethical principles that are intended to govern their application. Frameworks of principles include the Ethics Guidelines for Trustworthy AI by European Commission et al. (16) and the Principles for Responsible Stewardship of Trustworthy AI by OECD (17). Indeed, ethical frameworks abound; Mittelstadt (18) found that at least 63 public-private initiatives had produced statements describing high-level principles related to ethical AI, and the number has surely grown since then.

Ethical principles are only as good as their implementations. Audits, and particularly algorithmic audits are increasingly being used to understand whether ethical principles are in fact adequately implemented in practice. A number of auditing frameworks have been developed, such as the End to End Framework for Internal Algorithmic Auditing by Raji et al. (19).

However, all such audits suffer from the same challenge—namely, their post-hoc nature. This is particularly problematic when audits discover problems that arise from choices made, unconsciously or otherwise, at the early stages of creating a product or algorithm. With respect to the implementation of ethical principles, the biggest challenge is that ethical principles can exist in tension with each other, such that trade-offs must be made on how much to follow one principle at the expense of another. A pertinent example is the trade-off between privacy and avoiding bias. In Clavell et al. (15), some of the authors of this paper found, through an algorithmic audit, that an overemphasis on data minimization can in practice hinder efforts to avoid bias. This is because the goal of data minimization means that data relevant to understanding bias, such as gender, age, ethnicity, etc. is not collected, thereby forcing auditors to rely on indirect evidence of bias.

To avoid *driving by looking in the rear-view mirror*, step-by-step guides are required that allow the potential for bias to be considered right from the conception of a product or algorithm, through to its deployment and, of course, auditing.

### 2.2. About *Foundations*

*Foundations* is a mental wellbeing app, available on iOS and Android, with interactive, evidence-based programmes and activities to help users build resilience and manage stress. *Foundations* is offered as a Business-to-Consumer (B2C) product to employers as part of their Employee Assistance Program. It is designed to help work organizations support their teams, enabling people to take care of their mental wellbeing on their own terms in a cost-effective manner. Employees interact with the app, its programmes and activities to build resilience. The areas of focus are anxiety, depression, stress and trouble sleeping. The efficacy of *Foundations* was previously evaluated in a randomized control trial, where the intervention group (n=62) showed significant improvements compared to the control group (n=74) on measures of anxiety (GAD-7 score), resilience (CD-RISC score), sleep (MISS score), and mental well-being (WHO-5 score) within 2 weeks of using *Foundations*, with further improvements emerging at week 4 (20).

**Figure 1:**
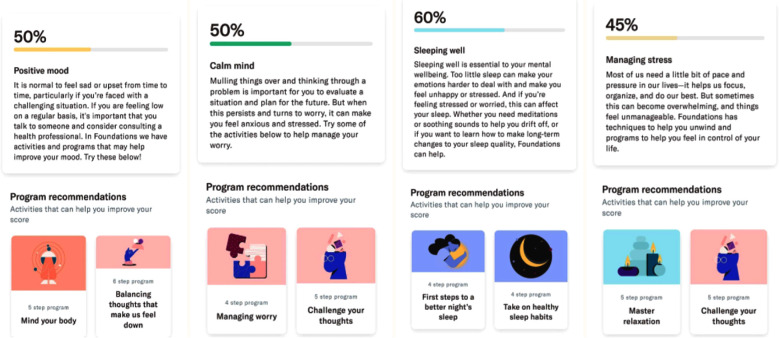
*Foundations* app screenshots.

*Foundations* offers programmes to help improve mental wellbeing through several activities (i.e., units of content): “Relax and unwind,” “Sleeping,” “Challenging negative thoughts,” “Positive thinking” and “Boosting self-esteem.” Activities are either *in the moment* (can be accessed at any time) or part of a *programme* (can be accessed only through the programme). The programmes are locked sequences of activities, delivered in daily steps designed to teach a skill, for example, to teach healthy sleep behaviours. In total, the app offers over 150 different activities to help the users manage their stress. Figure 1 illustrates sample activities and programmes included in *Foundations*.

To improve its engagement levels, *Foundations* recommends activities that users should explore outside of their active programs. For the current model version, the recommendations are presented in the *Today explore* widget. We further refer to this recommendation engine as the *Today explore* RecSys. The recommendation engine is based on the popularity of different activities during certain hours, depending on whether it is day or night. The recommendations are optimized for clickthrough rate by showing the user activities that the engine deems most relevant. The app has gone through several iterations since this analysis; the one here analyzed is the version from March 2021. The model training takes into account all the impressions and clicks. However, no data on gender, age, or other protected characteristics is used for training nor collected for *Foundations* everyday users as part of data minimization since this data is not needed for the functioning of *Foundations*.

#### 2.2.1 Risks of bias in *Foundations*

Within the focus of *Foundations* on mental health at a subclinical level, the main class of tasks is information access tasks such as search and recommendation. In particular, in the *Today explore* area of the app, users can access activities recommended to them, based on a mix of app usage and contextual data, such as the time of the day.

While developing *Foundations* we need to evaluate the process of monitoring or profiling users for bias (e.g., determining a health risk score) and then potentially provide this information to other algorithms, such as a decision support process as part of a dosage or intervention delivery mechanism. In a future extension of the app, we aim to passively monitor the severity of symptoms of users through models trained on smartphone sensing data. Undesired bias in categorization could affect a group of users who receive the wrong kind of care, as the algorithm can make inappropriate decisions based on biased or low-quality information. Potential issues in this task include providing less dosage or improper interventions to a group of individuals who need it the most when compared to others based on a protected attribute (such as disability status or gender). Depending on the algorithm design, such profiling could be, to some extent, caused by proxies for protected attributes.

Furthermore, undesired bias in recommendations could mean that some users do not receive any benefit from an activity or have adverse effects due to this activity. *Foundations*’ recommendations include suggestions for changing habits, behaviors, or the development of specific practices to improve users’ health. These have contextual implications from a societal standpoint, and in addition to having no benefit or being harmful, they might also reinforce stereotypes or stigmas.

#### 2.2.2 Sources of bias in *Foundations*

There are several potential sources of bias, including training datasets that encode past discrimination, or the actual design of models containing undesired bias, sometimes due to developers’ failure to acknowledge, or their unawareness of, issues of structural discrimination (21). Considering *Foundations*’ main goals, two primary sources of bias should be considered:

• *Expert-provided data*: Expert-provided data includes experts’ categorizations or rankings. These experts may be professionals in a relevant area or trained data annotators. Expert-provided data must be examined for categorizations that are strongly dependent on protected attributes or rankings that routinely place some items above others in an unjustified manner.
• *User-provided training data*: User-provided data includes explicit feedback as well as observed interactions (behavioral data) obtained from end-users in the operation of a system, including passive collected data. Systems trained on user-provided data containing undesired biases are susceptible to reinforcing biases existing in society or predominant within target groups. In a recommender system such as the one developed in *Foundations*, explicit feedback may be obtained from surveys, and behavioral data may include clicks or ratings of recommendations. Unwanted bias may be present in behavioral data if, for instance, a majority group of users (e.g., younger users) always rejects some recommendations that are actually very beneficial for a minority group of users (e.g., older users)—in this case, the minority group may experience less relevant recommendations.

### 2.3. 7 step process to assess fairness in automated systems

We leverage a step-by-step framework and present below the processes to assess fairness in automated systems, focusing on algorithmic bias detection. General recommendations introduce decisions to be made in order to properly conduct risk assessments and use appropriate methods at each stage of the process.

#### 2.3.1. Step 1: Contextual analysis

Establishing a solid basis for the analysis of algorithmic processing requires revisiting the rationale and theories behind the model design. This analysis includes aspects such as: (a) intended use and purpose of a (sub)system: determining users’ characteristics, categorizing users or items, generating recommendations, etc.; (b) theoretical basis of the model: are all variables or drivers associated with the target outcome captured adequately, considering the literature about the phenomenon? (c) fit-for-purpose features: are features appropriate for characterizing people from all groups? Does the system need to process more or different data from women, people with disabilities, or other groups? Is there any data or feature concerning a protected group which is missing? (d) characteristics of the ground-truth: is the ground truth an objective, physically measurable quantity, or does it contain some subjective elements? Is it obtained directly by observation or via inference? Is the ground truth the “real” target, or a proxy chosen for convenience? (e) completeness of training data: whether different groups are well represented in training data, particularly minorities. As a result of this examination, initial hypotheses about potential biases involving protected groups may be stated.

#### 2.3.2. Step 2: Mapping the user population

At the initial stage of development, it is also crucial to identify which protected groups might be at risk of bias. Therefore, the social context where the system will be used needs to be examined both within its envisioned context of use as well as with respect to its training data, if available. This includes a description of its targeted population at a sufficient level of detail in order to understand which protected groups it contains, including data on age-sex groups, age cohorts or other relevant factors (ethnicity, nationality, education level, etc.).

#### 2.3.3. Step 3: Prioritizing protected groups

The third step starts with identifying a reference group, which can be a structurally privileged or majority group (e.g., male, white). Next, protected groups within the population identified in Step 2 can be identified based on attributes or intersections of attributes. This includes particularly vulnerable groups (e.g., children with disabilities), large minority groups, women, and groups of particular concern with respect to the specific application at hand. The choice of protected groups and intersections of groups to analyze depends on the context and purpose of the algorithm. Potential harms (or lack of benefit) for disadvantaged groups must be hypothesized considering model outcomes and expected results since absolute algorithmic fairness measures will focus on differential effects of treatments between the protected and unprotected groups. Note, resource limitations may mean that we will not be able to prioritize all groups that we might wish to or need to undertake analysis in series. Such trade-offs should be described.

#### 2.3.4. Step 4: Selecting algorithmic fairness metrics

This step consists of choosing the most suitable metric for measuring identified disparities or potential adverse outcomes of the system regarding disadvantaged groups.

In theory, it could be possible to undertake a cost-sensitivity analysis in which each deviation from perfect parity is given a cost in arbitrary units or even in monetary ones. For instance, each additional percentage point of false negative rate disparity against a group might be equivalent to two additional percentage points of false positive rate disparity against another. However, in most cases, there is no reference point for performing this cost-sensitive assessment and no clear justification for the chosen costs. Hence, a possible hierarchy of metrics, in which some algorithmic fairness metrics are considered more important than others, can only be achieved in practice in a broad qualitative sense, if ever.

An additional task on this step is to determine the level at which the metric will be measured. For instance, a metric such as “satisfaction” can be computed at the level of the entire app (e.g., via a survey) or at the level of a specific recommender system (e.g., by observing whether users accept or do not accept the recommendations by that system).

#### 2.3.5. Step 5: Calculating the selected algorithmic fairness metrics

Various tools are available for this purpose, two popular tools are described next. Aequitas, an open-source toolkit of the Chicago University, is easy to use and includes a web-based tool to generate a report, configure bias metrics of interest and reference groups. It also has a Python Library to calculate bias and fairness metrics on data and predictions. Another tool is IBM AI360, a more feature-rich tool that includes methods for generating classifiers that satisfy algorithmic fairness criteria, usually at the cost of small decreases in terms of accuracy.

#### 2.3.6. Step 6: Analysing results, interpret using qualitative information

Identified differences for applied metrics between groups must be examined. Results should be checked against initial hypotheses, including the usual culprits such as training data representativeness or appropriateness of features for different groups. Some disparities could be justified through a careful application of, for instance, “business necessity”^3^ or another normative framing (23, 24). Other disparities may provide an advantage to a disadvantaged group and might not be as troubling as cases where a disadvantaged group is negatively impacted.

It should be noted that disparities in AUC or false negative rates are expected and fairly common in most deployed recommender systems. Quantitative results must be appropriately placed within the overall qualitative analysis to decide in which cases an action is necessary. To facilitate decisions on possible mitigation actions, the following warning levels are suggested: (1) *Most severe*: The analyzed algorithm or system harms a group or has no beneficial effect on a group who may be in harm’s way. (2) *Intermediate severity*: The algorithm or system has a positive effect but is substantially less effective, either in terms of performance or errors, for a vulnerable group (e.g., people with disabilities) or for a large group (e.g., women, people under 25 years old). (3) *Least severe*: The algorithm or system fails with respect to some algorithmic fairness criterion between a protected group and the reference group, or between two protected groups, however the disparate impact is relatively small. Whether a disparate impact (such as a discrepancy is false positive rates between two groups) is large or small, needs to be defined contextually within a specific application and with respect to specific groups, depending on factors such as how consequential the recommendations are and how vulnerable the group that experiences the disadvantage is.

#### 2.3.7. Step 7: Mitigating bias

Mitigation actions should be decided on the basis of severity, considering to what extent the criterion is violated and who the negatively impacted users are. Patterns of discrimination need to be identified, e.g., when both quantitative and qualitative analysis agree that the application has issues for some specific group.

In the case of risk assessment, learning models can be adjusted through in-processing changes, or their scores can be post-processed, or training data can be pre-processed (e.g., resampling, reweighting, or changing labels). This may lead to losses of accuracy that can be to some extent compensated with more training data, particularly for the group that exhibits less accuracy. It may also require additional features targeting specific characteristics of people in protected groups that can be good predictors of positive/negative outcomes for them.

Disparate impacts (Step 6) should be documented alongside the methods to mitigate them and any limits to mitigation efforts imposed by trade-offs with other goals, such as accuracy. Any remaining disparate impacts, where they affect end users, should be disclosed to them as limitations of a tool. For instance, if the app performs poorly for people over 65 and training data for that group is scarce, and/or for some reason that group is not within the scope of the app, the app should not be marketed to that group and a warning of this limitation should be made clear to potential users.

### 2.4. A randomized control trial

The primary aim of the RCT was to evaluate the efficacy of *Foundations* in improving the mental wellbeing during the COVID-19 pandemic, after 2 and 4 weeks of usage.

A 4-week RCT randomized controlled trial (RCT) was conducted which explored psychological and social wellbeing measures for London School of Economics students. Two apps were used in the trial, *Foundations*, a mental wellbeing app with interactive activities and programmes designed to build resilience, manage stress and improve sleep and LSEasy, an app designed to measure experiential subjective wellbeing.

Upon entry into the trial, all students were randomized to one of four groups: (1) *Foundations*, (2) LSEasy, (3) *Foundations* + LSEasy, or (4) control. Wellbeing measures were collected at baseline and weeks 2 and 4.

Participants were randomized individually with equal allocation to the 4 arms, stratified by gender (male, female, or other) and baseline WHO-5 score (≤12 or >12), using a random permuted block design.

The distribution of participants per arm, including the partial counts of participants for each sensitive attribute in Table 1, is shown in Table 2.

**Table 1:** Sensitive attributes analysed.

Sensitive attribute	Values
Gender	Female, Male
Working position	Do not work, Entry level, Internship
Employment status	Unemployed (not searching for job), Unemployed (searching for job), Employed
Location of origin	South and East Asia (incl. India and China), UK, Other Western Europe
Age	18–19, 20–26
Level of work	Full time, Part time, Other
Monthly income	<£1,000, £1,000–£2,000

**Table 2:** Distribution of participants per arm, total and for each value of sensitive attribute.

Sensitive attribute	Attribute value	Group 1	Group 2	Group 3	Group 4	Total
		153	153	151	153	610
Gender	Female	105	106	105	106	422
Gender	Male	46	46	45	45	182
Working position	Do not work	81	85	77	84	327
Working position	Entry level	25	19	20	16	80
Working position	Internship	19	20	20	22	81
Location of origin	South and East Asia	43	48	35	40	166
Location of origin	UK	42	48	49	45	184
Location of origin	Other Western Europe	24	23	26	31	104
Age	18–19	17	20	24	19	80
Age	20–26	112	105	97	112	426
Level of work	Full time	28	32	29	32	121
Level of work	Part time	60	54	51	51	216
Level of work	Other	62	67	68	67	264
Monthly income	<£1,000	110	121	115	116	462
Monthly income	£1,000-£2,000	28	26	22	25	101
Employment status	Unemployed (searching for job)	48	56	49	48	201
Employment status	Unemployed (not searching for job)	32	33	38	36	139
Employment status	Employed	44	42	41	38	165

Participants were paid £30 upon completion of the trial. Participants who were assigned to a group that included the use of *Foundations* were offered access to the app for free. Those in group 1 were considered to have completed the trial if they completed at least one programme and four activities in *Foundations*, and filled in both the onboarding and exit questionnaires. Participants in group 2 were considered to have completed the trial if they answered at least 70% of questionnaires and filled in both the onboarding and exit questionnaires. Participants in group 3 had to complete the completion requirements of both group 1 and group 2. Participants in group 4 were required to answer only the onboarding and subsequent check-up questionnaires at week 2 and 4 of the trial.

Participants were recruited from London School of Economics between March and April 2021. Upon the apps installation, they were first presented with a consent form detailing the objective of the RCT and data collected (in compliance with the GDPR regulations). The trial was reviewed and approved by the London School of Economics Ethical Board. Moreover, participants had to agree to a privacy policy for the onboarding questionnaire.

The pre-registration of the RCT can be found in https://osf.io/hvtf8.

## 3. Results

### 3.1. Evaluating fairness in *Foundations*’ effectiveness

#### 3.1.1 Step 1: Contextual analysis

*Foundations* is an application designed to be marketed to large organizations for their employees. Organizations licensing the app would provide it to their employees, which means that our target groups included adults who are employed full-time and are between 18 and 66 years of age. As such, some groups of users are explicitly excluded from using *Foundations*, such as the unemployed, school-aged children or students, and the retired. Within the scope of employees in the US and UK, there is a vast variety, although businesses in the following sectors are more likely to be buyers of *Foundations*: healthcare, education (teacher not students), finance, telecommunications, and industrial organisations. Nevertheless, in the study conducted, we enlarge the population characteristics to evaluate differential impact across extended protected groups.

#### 3.1.2. Step 2: Mapping the user population

Given the study use case of *Foundations*, we analyzed its impact across the following protected characteristics, age, gender, income and employment attributes, and location of origin. The relevant arms for this analysis were *Foundations* and *Foundations* + LSEasy (groups 1 and 3, as defined in Section 2.4).

#### 3.1.3. Step 3: Prioritizing protected groups

In *Foundations*, women were identified as the protected group for the bias analysis. This decision is based on the contextual analysis conducted within step 1 framing women as a potential disadvantaged population regarding *Foundations*. Beyond gender, we analysed the sensitive attributes presented in Table 1, for the values reported by at least 10% of the participants.

#### 3.1.4. Step 4: Selecting an algorithmic fairness metrics

Since *Foundations* is a mental wellbeing app, designed to help people take care of their mental wellbeing on their own terms, we are interested in measuring users’ satisfaction as measured by the progress in their mental wellbeing (WHO-5 score).

The following measures are used to monitor users’ progress in wellbeing during the usage of *Foundations*:

• *Step-up over 4 weeks*: WHO-5 scores can be categorized as corresponding to low (<28), regular (≥28, <50) or high (≥50) wellbeing levels. The values reported at on-boarding and 4 weeks later are compared and those participants that decreased at least one level are deemed to have stepped up (e.g. regular wellbeing at on-boarding and low wellbeing 4 weeks later).
• *Increment over 4 weeks*: An increment in wellbeing over 4 weeks has occurred when there is an increase of more than 10 points in the WHO-5 score.

We consider that there is no disparate impact in *Foundations*’ effectiveness when the probability of a user stepping up or having an increment is similar across protected groups.

#### 3.1.5. Step 5: Calculating the selected algorithmic fairness metrics

To assess bias in the metrics described in Step 4 we use Fisher’s exact test on contingency tables where the participants are split both by their metrics score and whether or not they belong to a protected group.

#### 3.1.6. Step 6: Analysing results

For each one of the metrics (step-up and increment) and each value of a sensitive attribute reported in Table 1 a contingency table was calculated as described in Step 5 and the Fisher’s exact test was applied.

For the gender attribute, the contingency tables related to the step-up and increment metrics are reported in Tables 3 and 4, respectively, and the corresponding Fisher’s exact tests yielded p-values of 0.359 and 0.169, respectively. We observe a large gender bias, which corresponds to an odds ratio of 1.80 for WHO-5 step-ups and 0.54 for WHO-5 increments between females and not females; however, the detected bias was not statistically significant.

**Table 3:** Contingency table for WHO-5 step ups (in the original data), split by gender values.

	WHO-5 step up	
Gender	Yes	No
Female	7 (8.23%)	78
Not female	6 (13.95%)	37

**Table 4:** Contingency table for WHO-5 increments (in the original data), split by gender values.

	WHO-5 increment	
Gender	Yes	No
Female	33 (38.82%)	52
Not female	11 (25.58%)	32

**Figure 2:**
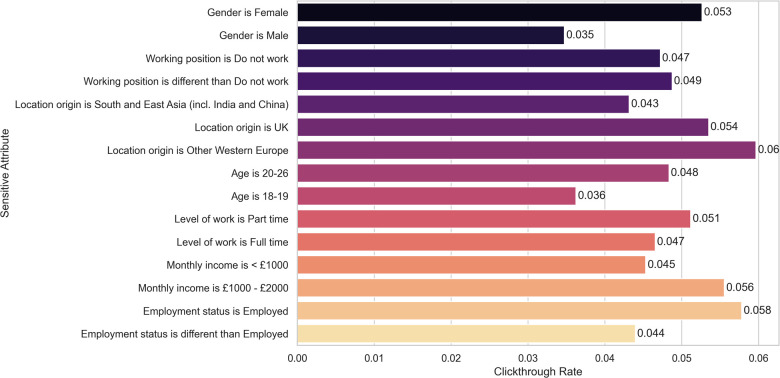
Percentage of WHO-5 step-ups for each sensitive attribute. None of the differences remain statistically significant after correcting for multiple hypotheses testing.

In summary, the (minimum) p-values for the Fisher’s exact tests for each sensitive attribute and metric (step-up and increment) are shown in Table 5, line 1 and 2, respectively. We illustrate the percentage of WHO-5 step-ups, defined as the number of step-ups divided by the total number of participants in that category, for each sensitive attribute in Figure 2, given its importance in clinical usage, namely in triage.

**Table 5:** Minimum *p*-values for Fisher’s exact test on contingency tables involving a sensitive attribute and the following targets: WHO-5 step ups in the original data, WHO-5 increments in the original data, and WHO-5 step up events as predicted by the step up model.

	Sensitive attribute						
	Gender	Working position	Employment status	Location of origin	Age	Level of work	Monthly Income
WHO-5 step up							
Minimum p-value	0.346	0.258	0.033${}^{*}$	0.213	0.059	0.546	0.690
(original data)							
WHO-5 increment							
Minimump-value	0.169	0.292	0.171	0.031${}^{*}$	0.011${}^{*}$	0.200	0.614
(original data)							
WHO-5 step up							
Minimum p-value	0.091	0.549	0.049${}^{*}$	0.049${}^{*}$	0.112	0.517	0.338
(step up model)							
Today explore							
Minimum p-value	0.035${}^{*}$	0.399	0.102	0.270	0.343	0.613	0.257
(RecSys model)							

Statistically significant p-values are marked with *.

While three of the statistical tests yielded statistically significant results (p-value <0.05) we need to take into account that multiple tests were performed (18 for each of the targets, i.e., WHO-5 increment and step-ups) and correct for multiple hypotheses. Using the correction by Benjamini-Yekutieli (25) we conclude that, after correction, none of the results is statistically significant.

#### 3.1.7. Step 7: Mitigating bias

No mitigation actions were further taken, given that the results from Step 6 were not statistically significant.

### 3.2. Use case results: Preliminary analysis on the disparate impact of step-up monitoring models in WHO-5

Continuous monitoring of users’ mental health state is a pre-requisite for delivering the right intervention at the right time. Asking users to frequently report their mental health states is not sustainable, which is the area where passive detection of symptoms can provide a breakthrough. Smartphone sensor data provides a proxy to everyday behaviours, such as diurnal patterns, sleep, mobility, physical and social activities—all correlated with mental health symptoms. Based on such data, we developed a machine learning model to detect important changes in wellbeing, reported by using the World Health Organization’s Well-Being Index. The 5-item World Health Organization Well-Being Index (WHO-5) is a short self-reported measure of current mental wellbeing (26). WHO-5 scores range between 0 and 25. They are rescaled to 0–100 and the following intervals are used to classify the scores into levels of wellbeing: *Low wellbeing*: [0, 28), *Regular wellbeing*: [28, 50), and *High wellbeing*: [50, 100].

WHO-5 questionnaire results taken at two different points in time are compared per individual as follows: *“step-up”* if the second result is in a higher severity level than the first one (equivalent to a lower score in WHO-5, denoting a deterioration in wellbeing); *“no change”* if both results are in the same severity level; and *“step-down”* if the second result is in a lower severity level than the first one (equivalent to a higher score in WHO-5, denoting an improvement in wellbeing). Note that this includes the following cases: (a) when a user steps up by one level between the two points in time (e.g., from regular to low); or (b) when a user steps up by several levels between the two points in time (e.g., from high to low).

The intended use of the algorithm is to passively monitor wellbeing outcomes and detect deterioration in wellbeing level. Enabling a continuous assessment of the user state would open the door to changing the mental healthcare paradigm towards the continuous and stepped-care model—delivering appropriate interventions timely and with a proper dosage, and ultimately improving health outcomes.

We utilize the gradient boosting library called XGBoost to train a machine learning algorithm to predict step-up events of deterioration in WHO-5, according to standard labelling of low, regular and high at the two different points in time. We compare its performance against two other machine learning models, namely Logistic Regression and Random Forest (using n=500 trees), using the following metrics: Area Under Curve for the receiver operating curve (AUC), sensitivity, specificity, precision, recall, balanced score and Kappa score. We utilized a mixture of tree-based and linear models to explore models of different complexities, with the tree-based ones having a low bias and high variance, while the regression has a high bias and low variance. The model that performs best across the majority of the evaluation metrics is the XGBoost one. The models were trained using passively collected data, which was transformed into features that capture the variations between the individual’s behaviour between two different moments in time. These features are designed to reflect behaviour and cognitive state changes between the start of the period and the end of the period. In that sense, the data was aggregated at a daily level over the period of time that the system is meant to detect the state change, such as average across days, total sum, standard deviation across days, variance across days, minimum value, etc. In the second stage, the set of variables is transformed into the change-based features that are used in the following step for modeling (e.g., similarity between the mean number of steps during week 1 and 2, compared to week 3 and 4).

#### 3.2.1. Step 1: Contextual analysis

The intended use of the step-up model is to function as a triage model with the purpose of detecting users that decrease their well being significantly, and hence, enable a stepped care model where the user is given the next level of care, within their consent. For instance, a different mental health app may be prescribed to them, or the therapist may be notified about the deterioration, should this be within the scope of the app and their consent. The ground truth of the stepped care model is computed based on a threshold obtained by literature. WHO-5 score was self-reported by the users.

#### 3.2.2. Step 2: Mapping the user population

As in the analysis of *Foundations*’ effectiveness the protected characteristics that we will pay attention to are: age, gender, income and employment attributes, and location of origin. We utilized data coming from users who had installed the LSEasy app for passive monitoring, since we build the predictive model based on passive signals from the phone. This includes users who installed only the LSEasy app, as well as LSEasy and *Foundations* apps (groups 2 and 3, as defined in Section 2.4.

#### 3.2.3. Step 3: Prioritizing protected groups

As in the analysis of *Foundations*’ effectiveness, women were identified as the protected group for the bias analysis. Beyond gender, we again analysed the sensitive attributes presented in Table 1, for the values reported by at least 10% of the participants.

#### 3.2.4. Step 4: Selecting an algorithmic fairness metrics

We consider that the model for monitoring a user’s state is fair when the probability of stepping up is similar across protected categories. Therefore, we utilized Fisher’s exact test to compare the probability of stepping up is statistically significant across protected categories.

#### 3.2.5. Step 5: Calculating the selected algorithmic fairness metrics

We performed a pairwise Fisher exact two-tailed test for subgroups within each category. Given the small sample size we decided not to create new subgroups out of the protected attributes (e.g. Male with an income of <£1,000).

#### 3.2.6. Step 6: Analysing results

We present the results of the step-up model in Table 6. Moreover, for each protected attribute, we computed the contingency table related to the step-up events detected and the corresponding Fisher’s exact test p-values. In summary, the (minimum) p-values for the Fisher’s exact tests for each sensitive attribute are shown in Table 5, line 3.

**Table 6:** Step up in WHO-5 level (e.g. regular to low = Step up) with a machine learning model.

Model	Confusion matrix	AUC	Sensitivity	Specificity	Precision	Recall	Balanced score	Kappa
XGBoost step up model	[47, 6], [7, 5]	0.66	0.42	0.89	0.45	0.42	0.65	0.31
Logistic regression step up model	[47, 6], [8, 4]	0.69	0.33	0.89	0.40	0.33	0.61	0.24
Random forest step up model	[50, 3], [10, 2]	0.74	0.16	0.94	0.40	0.16	0.56	0.14

While two of the statistical tests yielded statistically significant results (p-values <0.05), their value was at the border of significance (0.049), and we need to take into account that multiple tests were performed (18 per metric) and correct for multiple hypothesis. Using the correction by Benjamini-Yekutieli (25) we conclude that, after correction, none of the results is statistically significant.

#### 3.2.7. Step 7: Mitigating bias

No mitigation actions were further taken, given that the results from Step 6 were not statistically significant.

### 3.3. Use case results: Preliminary analysis on the disparate impact of *Today explore* RecSys

#### 3.3.1. Step 1: Contextual analysis

The intended use of the *Today explore* Recsys is to improve the engagement levels in *Foundations* by providing better activity recommendations. In particular, it recommends activities that users should explore which are outside of their active programs. For the version of *Foundations* used during the study (3.2.0), the recommendations are presented in the *Today explore* widget.

The recommendation engine is based on the popularity of different activities during certain hours, depending on whether it is day or night. This model aims to optimize for clickthrough rate on activities, by showing the user activities that it classifies as most relevant. No data on sensitive attributes is used for training nor collected for *Foundations*’ everyday users as part of a data minimization strategy since this data is not needed for the functioning of *Foundations*.

#### 3.3.2. Step 2: Mapping the user population

As in the analysis of *Foundations*’ effectiveness, the protected characteristics that we will consider are: age, gender, income and employment attributes, and location of origin. We selected the study participants from the *Foundations* and *Foundations* + LSEasy arms (groups 1 and 3, as defined in Section 2.4) who completed at least one programme and four activities in *Foundations* (n=219).

#### 3.3.3. Step 3: Prioritizing protected groups

As in the analysis of *Foundations*’ effectiveness, women were identified as the protected group for the bias analysis. Beyond gender, we again analysed the sensitive attributes presented in Table 1, for the values reported by at least 10% of the participants.

#### 3.3.4. Step 4: Selecting an algorithmic fairness metrics

*Today explore* Recsys was designed with the goal of improving engagement levels in *Foundations* through additional clicks in the *Today explore* widget. Therefore, a key indicator to evaluate its performance is the click-through rate, measured as the ratio between activities selected and activities recommended.

As a fairness metric we use the related binary variable which takes the value 1 when a user selects the recommendation and 0 when a user does not select it. The click-through rate can be computed as the average value of this metric.

#### 3.3.5. Step 5: Calculating the selected algorithmic fairness metrics

To assess bias in the fairness metric described in Step 4 we use Fisher’s exact test on contingency tables where recommendations are split by the fairness metrics value and whether they were shown to a user with the relevant sensitive attribute or not.

#### 3.3.6. Step 6: Analysing results

For each value of a sensitive attribute reported in Table 1, a contingency table was calculated as described in Step 5 and the Fisher’s exact test was applied.

**Figure 3:**
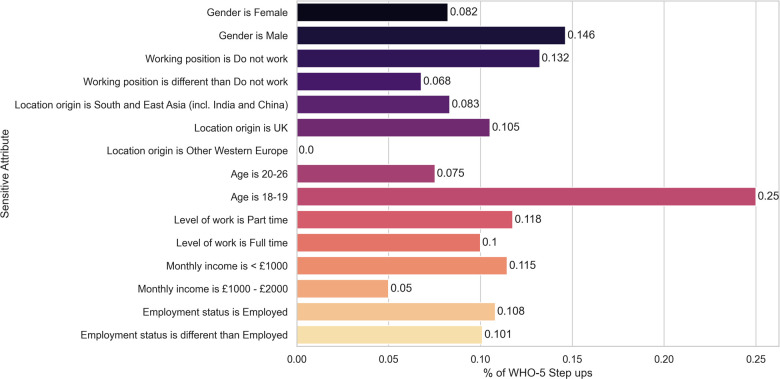
*Today explore* RecSys Clickthrough rate for each sensitive attribute. None of the differences remain statistically significant after correcting for multiple hypotheses testing.

In Table 7 we show the contingency table related to the fairness metrics for the gender attribute. The corresponding Fisher’s exact test yielded a p-value of 0.035, with female participants benefiting more than male participants from the recommendations of *Today explore* Recsys. In summary, the (minimum) p-values for the Fisher’s exact tests for each sensitive attribute are shown in Table 5, line 4. We illustrate the clickthrough rates for each sensitive attribute in Figure 3.

**Table 7:** Contingency table for Today Explore Recsys outputs, split by gender values.

	Activity selected		
Gender	Yes	No	Clickthrough rate
Female	128	2,303	5.27%
Not female	27	762	3.42%

While the statistical test for female participants yielded a statistically significant result we need to take into account that multiple tests were performed (in particular 18 tests) and correct for multiple hypothesis. Using the correction by Benjamini-Yekutieli we conclude that, after correction, the result is not statistically significant.

#### 3.3.7. Step 7: Mitigating bias

No mitigation actions were further taken, given that the results from Step 6 were not statistically significant.

## 4. Discussion

As digital mental health services are expanding, it is becoming increasingly important to understand fairness in the provision of those services. Existing literature provides little guidance on this topic in the domain of digital mental health services. In this paper, we shed light on the process of auditing digital mental health services and their algorithms as an integral part of digital mental healthcare delivery. We advocate for a greater focus on fairness analysis in this domain in which sensitive user groups (based on age, gender, ethnicity, etc.) may be impacted differently by mental health services and their embedded automated systems.

### 4.1. Overall *Foundations*’ impact in wellbeing

At the overall app level, we found that there is no difference when analysing four of the protected groups: gender, working position, level of work and monthly income. In the other three groups (i.e., employment status, age, and location of origin), we did find some small differences regarding *Foundations* efficacy, but only before correcting for multiple hypotheses testing. In the case of employment status, we found that the people who had selected “Unemployed (not searching for job)” (N=27) benefited less from the app, and had a higher percentage of step-ups (odds ratio: 0.266 compared to the rest; 22.22% in this category stepped up, compared to 7.07% for the rest). Moreover, in the case of “Age,” we found that people between 18 and 19 years of age (N=16) had less benefits from the app in terms of WHO-5 increments (odds ratio: 9.35; 6.25% of increments in this category, compared to 29.46%). The majority of people of this age included in the study were unemployed (62.5%). In the case of “Location of origin,” we found that people from “Other Western Europe” (N=17) benefited less from the app and had a lower rate of WHO-5 increments (odds ratio: 5.09, compared to the rest; 11.11% of increments in this category, compared to 38.88%). In this subgroup of participants 47% of them were unemployed. This is somehow expected and in line with an app that targets a working population. It is within reason to expect that unemployed people will benefit less than employed people within such a short time frame (4 weeks). Nevertheless, none of these results remained significant after correcting for multiple hypotheses.

### 4.2. Overall impact of the *Step-up* monitoring models

We found two results which were at the limit of statistical significance, since their p-values were 0.049, in the categories “Employment status” and ‘Location of origin.” In the case of “Location of origin,” we observed that the model was particularly accurate for people from South East Asia (N=18), correctly categorizing 17 out of 18 cases. This was the second largest group in our training sample. Moreover, this group had only 2 step-ups. Similarly, in the case of “Employment status,” we observed that the model was particularly accurate for people who were “Unemployed (searching for a job),” correctly categorizing correctly 17 out of 18 cases. Moreover, this group had only 1 step-up. The results are not surprising given that these groups represent a large portion of the training data and have a low number of step-ups, meaning that the majority of people did not deteriorate in well-being, which is easier for the model to learn. Nonetheless, none of the results remain significant after correcting for multiple hypotheses. In conclusion, in a potential future extension of *Foundations*, where the step-up model would be deployed for monitoring users’ wellbeing passively, we do not expect significant disparate impact in any of the seven studied salient groups.

### 4.3 Overall impact of the *Today explore* RecSys

The *Today Explore* recommender system is only a part of the *Foundations* app, and there are other elements of the app with which users interact. In particular, for this study, participants were required to complete at least one programme within *Foundations*, which may have limited the time they invested in other parts of the app. However, the recommender system is a specific element that we identified as having a potential risk of algorithmic bias and this is why we analyze it. Nonetheless, the click through rate for the RecSys was below 5% during this study, hence, having little impact on the overall engagement and efficacy. Nevertheless, we established a protocol for evaluating the algorithm fairness of the RecSys in the future, we assessed its current bias and found no statistically significant results after correction in any of the seven studied protected groups and finally, we are completing a model card that will be used before deploying models in production.

### 4.4. Difference between the study population and *Foundations*’ active users

Currently and in the foreseeable future, there is no plan to collect sensitive attributes from the active users of *Foundations* (e.g., gender or age) due to internal privacy policies within Koa Health. Therefore, we have less information about our users which in turn leads to fewer ways to personalize *Foundations*, and can also delay the discovery of bias against protected groups. For these reasons, it is difficult to compare the sample population from the study with the active users of *Foundations*. Nevertheless, we plan to discover biases when running randomized control trials with a large sample population.

### 4.5. Ethical considerations

The research presented in this paper has been reviewed against Koa Health’s ethical commitments in its Ethics Impact Assessment (EIA)^4^ framework. In terms of ethical concerns with respect to *Foundations*’ efficacy and the algorithms presented in the two use cases (WHO-5 step-up model, and RecSys), the most important are:

• Ensuring that there is a positive impact on users’ happiness, health and wellbeing.
• Avoiding biases that discriminate against protected groups.
• Maximising privacy of users’ personal data.
• Ensuring that the algorithm does not lead to users becoming addicted to *Foundations*.

The first three of the above points were all considered within the analysis on discrimination, intended use, subgroups, trade-offs and limitations.

Addiction is not considered to be a challenge at this stage in the maturity of the algorithm, nor of *Foundations* more generally. This is because the app does not use features that can lead to addiction, such as infinite scroll, social validation, etc.; and the algorithm does not support these. See the *Foundations* EIA for more details.

## 5. Conclusion

Mobile technologies and apps for mental self-care have became prominent in recent years. Socio-economic and gender biases have been identified in such systems, including digital divide problems caused by inequalities in access to digital services, and lack of consideration for gender differences. Moreover, some of these mobile technologies are powered by machine learning models, which can perpetuate existing biases and present risks of algorithmic discrimination. In this paper, we assess *Foundations*, a mental health app, that aims to help people deal with stress in the workplace, regardless of gender, race, ethnicity and other protected attributes, and by design should have no disparate impact, since it is built on evidence-based interventions meant to be effective for all. We argue that evidence-based tools still need to go through an ethics impact assessment and we cover the following evaluations in practice: (a) whether the content of *Foundations* has any disparate impact on protected groups, (b) whether a future extension of *Foundations* offering a passive monitoring service has any undesired bias, and (c) whether the existing version of RecSys at the time of the study had any undesired bias. In this study, we found no disparate impact and no undesired bias in our evaluations.

## Data Availability

The datasets presented in this article are not readily available because **the data used for this study can be shared only under a data sharing agreement**. Requests to access the datasets should be directed to aleksandar.matic@koahealth.com.

## Footnotes

^1^The liability and (un)intentional character of discrimination determines differences concerning its legal disparate treatment (deliberate) or impact (unintentional) over individuals (2).

^2^ https://foundations.koahealth.com

^3^For instance, as Raghavan et al. (22) explained, some vendors of algorithmic hiring assessments avoid disparate treatment solely by assuring that protected attributes such as race are not used as inputs to their models. However, with regards to disparate impact, vendors can still fail, and such limitations may be legal within some legal frameworks. Even if the screening algorithm does produce a disparate impact, it can be justified as assisting a legitimate business objective if it is accurate enough.

^4^ https://koahealth.com/ethics˙audit˙koa˙health˙apps.pdf
